# Bacterial endophytes as indicators of susceptibility to Cercospora Leaf Spot (CLS) disease in *Beta vulgaris* L.

**DOI:** 10.1038/s41598-022-14769-8

**Published:** 2022-06-23

**Authors:** Chiara Broccanello, Samathmika Ravi, Saptarathi Deb, Melvin Bolton, Gary Secor, Christopher Richards, Laura Maretto, Maria Cristina Della Lucia, Giovanni Bertoldo, Elena Orsini, María Gabriela Ronquillo-López, Giuseppe Concheri, Giovanni Campagna, Andrea Squartini, Piergiorgio Stevanato

**Affiliations:** 1 Department of Agronomy, Food, Natural Resources, Animals and Environment, University of Padua, Viale Dell’Università, Legnaro, PD Italy; 2 Northern Crop Science Laboratory, U.S. Dept. Agriculture, Fargo, ND USA; 3 Plant Pathology Department, North Dakota State University, Fargo, ND USA; 4 USDA ARS National Laboratory for Genetic Resources, Fort Collins, CO USA; 5 Strube Research GmbH & Co. KG, Söllingen, Germany; 6 COPROB, Minerbio, BO Italy

**Keywords:** Plant breeding, Sequencing

## Abstract

The fungus *Cercospora beticola* causes Cercospora Leaf Spot (CLS) of sugar beet (*Beta vulgaris* L.). Despite the global importance of this disease, durable resistance to CLS has still not been obtained. Therefore, the breeding of tolerant hybrids is a major goal for the sugar beet sector. Although recent studies have suggested that the leaf microbiome composition can offer useful predictors to assist plant breeders, this is an untapped resource in sugar beet breeding efforts. Using Ion GeneStudio S5 technology to sequence amplicons from seven 16S rRNA hypervariable regions, the most recurring endophytes discriminating CLS-symptomatic and symptomless sea beets (*Beta vulgaris* L.ssp. *maritima*) were identified. This allowed the design of taxon-specific primer pairs to quantify the abundance of the most representative endophytic species in large naturally occurring populations of sea beet and subsequently in sugar beet breeding genotypes under either CLS symptomless or infection stages using qPCR. Among the screened bacterial genera, *Methylobacterium* and *Mucilaginibacter* were found to be significantly (p < 0.05) more abundant in symptomatic sea beets with respect to symptomless. In cultivated sugar beet material under CLS infection, the comparison between resistant and susceptible genotypes confirmed that the susceptible genotypes hosted higher contents of the above-mentioned bacterial genera. These results suggest that the abundance of these species can be correlated with increased sensitivity to CLS disease. This evidence can further prompt novel protocols to assist plant breeding of sugar beet in the pursuit of improved pathogen resistance.

## Introduction

The fungus *Cercospora beticola* is the cause of a major disease in sugar beet called Cercospora Leaf Spot (CLS). The disease is most prevalent in humid and temperate zones of northern Italy, Greece, northern Spain, Austria, southern France, Japan, China, and United States of America (including Red River Valley region of North Dakota and Minnesota)^1^. The disease is initiated when conidia land on the leaf surface. Upon germination, hyphae penetrate the leaf surface through stomata and spread intercellularly^2^. The subsequent production of phytotoxins and degradative enzymes leads to the development of small necrotic lesions which, in the presence of favorable environmental conditions, expand and cover the entire leaf. Crop yield losses up to 50% are reported due to the damage to the foliar apparatus^3^.

CLS management is typically based on the cultivation of resistant varieties, fungicide treatments, and agronomic practices such as crop rotation. However, the breeding of tolerant hybrids is still a major challenge^4^. Genetic resistance involves the presence of numerous alleles with additive effects. Given the limited grasp on the key players mediating genetic resistance, the search for molecular markers relevant for breeding selection is an ongoing task^5^. Breeding programs for CLS have primarily focused on the diversity offered by the plant’s genome and not on clues that could be revealed while examining host-microbial associations^6^. With the beginning of the microbiome era, researchers are starting to appreciate that the genetic determinant of the host can affect both its fitness and the community of the microbes that live inside and on plant tissues^7^.

Sea beet, *Beta maritima (Beta vulgaris L. subsp. maritima)*, has commonly been utilized as a source of key traits because of its evolutionary proximity with the related cultivated species of *Beta vulgaris*. These wild populations grow spontaneously along the European coasts presenting high plasticity in response to abiotic stresses such as drought, high temperature, and salt tolerance and biotic stresses like CLS, rhizomania, and nematodes^8^. This permits the exploration of new biomarkers originating from the microbiome of sea beet and associated with resistance to adverse conditions.

Plants, as sessile organisms, need to evolve strategies to evade biotic and abiotic stresses. One of these strategies is their relationship with bacteria and fungi that can be positively exploited^9^. While the main source of microorganisms that reside in the plant microbiome originates from the soil, the microbiome profile of a plant is shaped by the rhizosphere, phyllosphere, and by microorganisms present in the air. When internalized as endophytes, these microbes ultimately constitute the so-called plant’s endosphere^10^. Plants release a variety of root exudates, organic acids, amino acids, sugars, and vitamins that are used by microbes as nutritional sources, and signals to enhance metabolic activities. Conversely, endophytes secrete phytohormones, small molecules, or volatile compounds that can be recognized by plants as general elicitors of plant defense^2, 11^. Molecules such as N-acyl-L-homoserine lactones, which belong to the quorum sensing microbial communication mechanism, are also sensed by plants and can regulate their gene expression^12^. Phytohormones like auxin and cytokinin increase water and nutrient uptake^13^ and enhance plant growth and development^14^. The root microbiome has also been shown to stimulate plant innate immunity, conferring resistance against leaf pathogens, a phenomenon known as induced systemic resistance^15^. This type of resistance appears to be conserved among organisms and early contact with microbial molecules is vital for plant survival^16^. Nevertheless, one should not assume that all microbes living inside a plant without apparent disease symptoms are beneficial. Many endophytes could also have other non-mutualistic behaviour, some could have no evident effect and others could be also deleterious. It is therefore important to consider that in principle, endophytes can have undefined roles in plant health that require thorough evaluation. For example, a recent study that tested isolates from potato plants in specific bioassays showed that many endophytes turned out to be plant growth-neutral (56%). The others were either plant growth-promoting (21%) or plant growth-inhibiting (24%)^17^.

The development of NGS technologies has significantly expanded our understanding of the composition and role of microbial communities. These culture-independent methods generate large amounts of data and favour the discovery of complex dynamics in host–pathogen diseases. Metagenomics studies, when focusing on bacteria using specific taxonomical metabarcoding, rely on the analysis of 16S ribosomal RNA sequences^18^. The 16S rRNA gene includes nine hypervariable regions V1-V9. Some of these regions are partly conserved at specific rank levels and thus used for broad lineage placement, while the more variable stretches allow for the identification of genera and species^19^. A series of experimental studies have shown that the choice of 16S rRNA region significantly affects the accuracy in the estimation of taxonomic diversity^20, 21^. It was shown that V2 and V4 regions have the lowest error rate in taxa identification compared to other regions, while Claesson et al.^22^ found the highest accuracy of classification using the V3 and V4 regions. Other studies have used the V6 region, demonstrating that it could underestimate the diversity of bacteria in the phyla *Verrucomicrobia* and *Bacteroidetes*^23^.

In this paper, we present sequencing results obtained using the Ion GeneStudio S5 technology and 16S Ion Metagenomics Kit which simultaneously amplifies seven hypervariable regions, V2, V3, V4, V6, V7, V8, and V9, of the bacterial 16S rRNA gene.

The overall goal of this study was to explore and correlate endophyte abundance as a predictive factor associated with susceptibility or resistance to CLS to exclusively assist selection in sugar beet breeding programs. To achieve this, as our first objective, we analysed the bacterial endophytic content of sea beet, the wild progenitor of sugar beet including both CLS-symptomatic and symptomless plants to determine the contrasting abundance of prokaryotic taxa. The second objective was to design an inexpensive method to survey the endophytic abundance in large naturally occurring sea beet populations along the Mediterranean coast. For this, we designed non-degenerate primers for the specific detection of bacterial species based on the 16S rRNA gene sequencing and to verify their distinctive presence on CLS-symptomatic or symptomless phenotypes. The last objective was to substantiate the quantitative association of significantly differentially abundant bacteria on a range of resistant and susceptible genotypes of cultivated beets.

## Results

### Bioinformatics analysis of sea beet microbiome

A total of 1,485,067 reads was obtained from the sequenced samples comprising four symptomless and four symptomatic sea beets. The mean number of reads per sample was 185,633 with a range of ± 49,918. Rarefaction analysis reporting the number of observed species across all samples indicated that the diversity was not significantly different between the sample groups evaluated using richness as a parameter (p = 0.97) (Supplementary Fig. S1). At 97% identity, the reads were classified into 85 OTUs further divided into 29 families, 22 genera, and 38 species using the Greengenes database v13.5^24^ and the curated MircoSeq reference library v2013.1 on the Ion Reporter cloud. Further, analysis using QIIME2^25^ resulted in 514 amplicon sequence variants (ASVs) which were classified into 35 orders, 47 families, and 70 genera. Evidently, QIIME2-based data analysis resulted in the classification of more genera. However, the classification and representation of major genera remained comparable between both the methods used (Supplementary Fig. S2). The outputs from QIIME2 were considered more robust and used for further elaboration.

**Figure 1:**
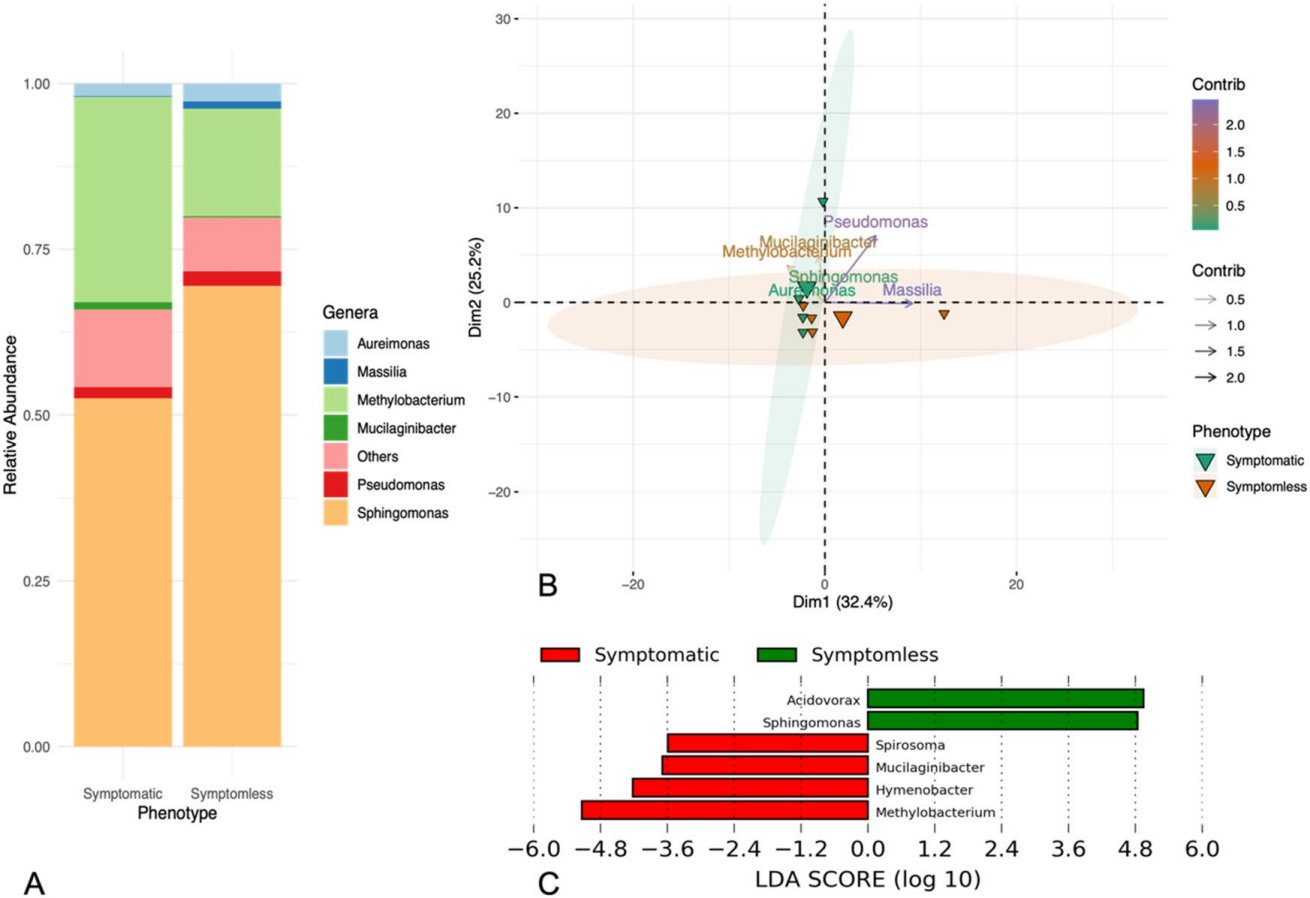
(**A**) Microbiome composition presented as relative abundances between symptomless and symptomatic sea beets. (**B**) Principal Component Analysis (PCA) biplot showing the variation among the sea beet phenotypes and the abundances of microbial genera. Individuals on the same side of a given variable have a higher value for the same. (**C**) Linear Discriminant Analysis (LDA) Effect Size (LEfSe) plot of taxonomic biomarkers. Positive LDA scores (green bars) are enriched in symptomless plants while negative LDA scores (red bars) are enriched in symptomatic plants.

The bacterial community of the sequenced *Beta maritima* leaves is presented with a relative abundance plot in Fig. 1A. The genus *Sphingomonas* was found predominant reaching a percentage of 69.4% in symptomless and 52.5% in symptomatic samples (Fig. 1A). Other predominating genera detected on symptomless and symptomatic samples were: *Methylobacterium* (16.2% and 31.0%)*, Aureimonas* (2.7% and 1.9%)*, Mucilaginibacter* (0.2% and 1.1%), *Massilia* (1.1% and 0.09%) and *Pseudomonas* (2.2% and 1.7%). Other genera encountered, but not as consistently and with minor abundances (< 1%), included *Spirosoma, Hymenobacter, Sphingobacterium, Roseomonas, Acidovorax, Nocardioides, Siphonobacter, Microbacterium,* and *Cupriavidus* (Supplementary Fig. S2)*.*

The contribution of the abundances of predominant bacterial genera (present > 1%) to the sample phenotypes is visualised as a PCA-biplot in Fig. 1B. The first two principal components explained 57.6% of the variability in the data. The arrows indicate the direction and strength of each variable to the overall distribution. A stronger positive correlation of *Methylobacterium* and *Mucilaginibacter* can be seen towards the symptomatic plants. In contrast, the abundances of *Pseudomonas* and *Massilia* are less correlated to the symptomatic phenotype. The commonly abundant genera, like *Sphingomonas* and *Aureimonas,* show the least contribution to the separation of the phenotypes.

Additionally, linear discriminant analysis (LDA) effect size (LEfSe) analysis revealed differences in the composition of the sea beet microbiome. The results indicated a higher enrichment of *Methylobacterium, Hyemenobacter, Mucilaginibacter,* and Spirosoma in symptomatic plants and a higher presence of Sphingomonas and Acidovorax in symptomless plants. The trends remain comparable between PCA-biplot and LEfSe analysis for *Methylobacterium* and *Mucilaginibacter (Fig. 1C and Supplementary Fig. S3)*.

### Validation of bacteria found associated with CLS on naturally occurring sea beet populations

The abundance of *Cercospora beticola* and of the most distinctively abundant bacteria between the sample groups presented in Fig. 1 were taken for downstream validation on larger sample sizes using Real-Time qPCR with QuantStudio 12 K Flex (Life Technologies, USA). In total 1,512 qPCR reactions were carried out on 504 independent sea beet individuals collected in the years 2019 and 2021 across coastal localities covering 3 countries (Table 1) in triplicates. The number of analysed symptomless and symptomatic plants were comparable, being 288 and 216 specimens respectively.

**Table 1:** Locations from which the 504 naturally occurring plant specimens of sea beet were collected.

ID	Location	Year of sampling	Number of symptomless samples	Number of
				symptomatic
				samples
1	Termoli (Italy)	2019	6	0
2	Vasto (Italy)	2019	7	1
3	Numana (Italy)	2019	46	34
	Numana (Italy)	2021	46	34
4	Porto Levante (Italy)	2021	15	12
5	Grado (Italy)	2021	70	25
6	Sečovlje (Slovenia)	2019	10	22
7	Ulica Istarkih (Slovenia)	2019	0	7
8	Sveti Ivan, Umag (Croatia)	2020	6	11
9	Ulica Slanik, Umag (Croatia)	2020	24	26
10	Novigrad (Croatia)	2020	13	5
11	Medulin (Croatia)	2020	19	8
12	Raša (Croatia)	2020	10	0
13	Labin (Croatia)	2020	3	4
14	Sveta Marina (Croatia)	2020	11	27
15	Koromačno (Croatia)	2020	2	0
	Total		288	216

**Figure 2:**
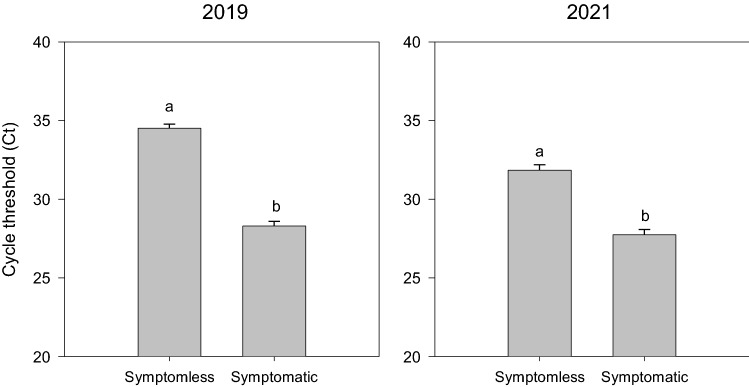
Relative abundance by qPCR of *Cercospora beticola* in symptomless and symptomatic sea beet individuals sampled in the year 2019 (left) and 2021 (right). Plots were generated using SigmaPlot version 11.0, www.systatsoftware.com).

The relative abundance of *Cercospora beticola* determined in symptomless and symptomatic sea beet individuals is depicted in Fig. 2. The fungus was detected in all samples irrespective of the phenotype but was found to be significantly higher in symptomatic sea beets for the years 2019 (p < 0.05) and 2021 (p < 0.05). To obtain a parallel insight, we used digital PCR allowing the maximum resolution and sensitivity with assays for *Cercospora* (Supplementary Fig. S4). Results confirmed the differences reported by qPCR. The correlation of abundance analyses with the two methods was significant (p < 0.05) for the same.

**Figure 3:**
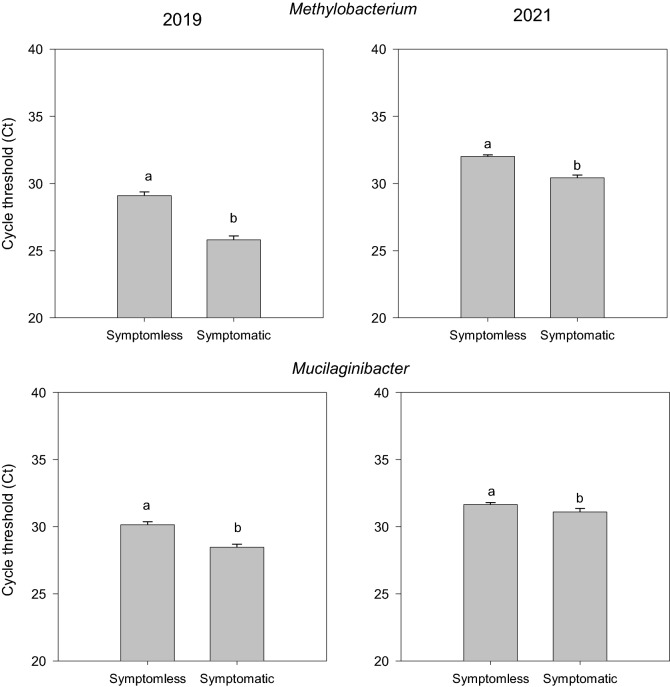
Relative abundance between CLS symptomless and symptomatic sea beet plants (p < 0.05) collected in the years 2019 and 2021 for (**A**) *Methylobacterium* and (**B**) *Mucilaginibacter* (generated using SigmaPlot version 11.0, www.systatsoftware.com).

Among the many bacterial taxa tested for which primer sequences are provided in Supplementary Table S1, *Methylobacterium* and *Mucilaginibacter* had a significant cycle threshold (Ct) variation (p < 0.05) revealed by qPCR analysis between symptomatic and symptomless samples (Fig. 3) across both the years. This was found to be consistent with the observations from sequencing presented in Fig. 1 and Supplementary Fig. S3, which resulted as the biomarkers enriched in symptomatic plants from LDA Effect Size (LEfSe) analysis. Particularly, the abundance of these bacteria was found to be significantly increased in the symptomatic plants. *Methylobacterium* genus showed an average Ct value of 29.1 ± 0.271 and 32 ± 0.12 in symptomless samples and 25.8 ± 0.291 and 30.4 ± 0.2 in symptomatic samples for the years 2019 and 2021, respectively. *Mucilaginibacter* showed an average Ct value of 30.1 ± 0.218 and 32 ± 0.13 in symptomless samples and 28.5 ± 0.226 and 31 ± 0.25 in symptomatic samples for the years 2019 and 2021 respectively.

### Validation of bacteria found associated with CLS on field-grown sugar beets

**Figure 4:**
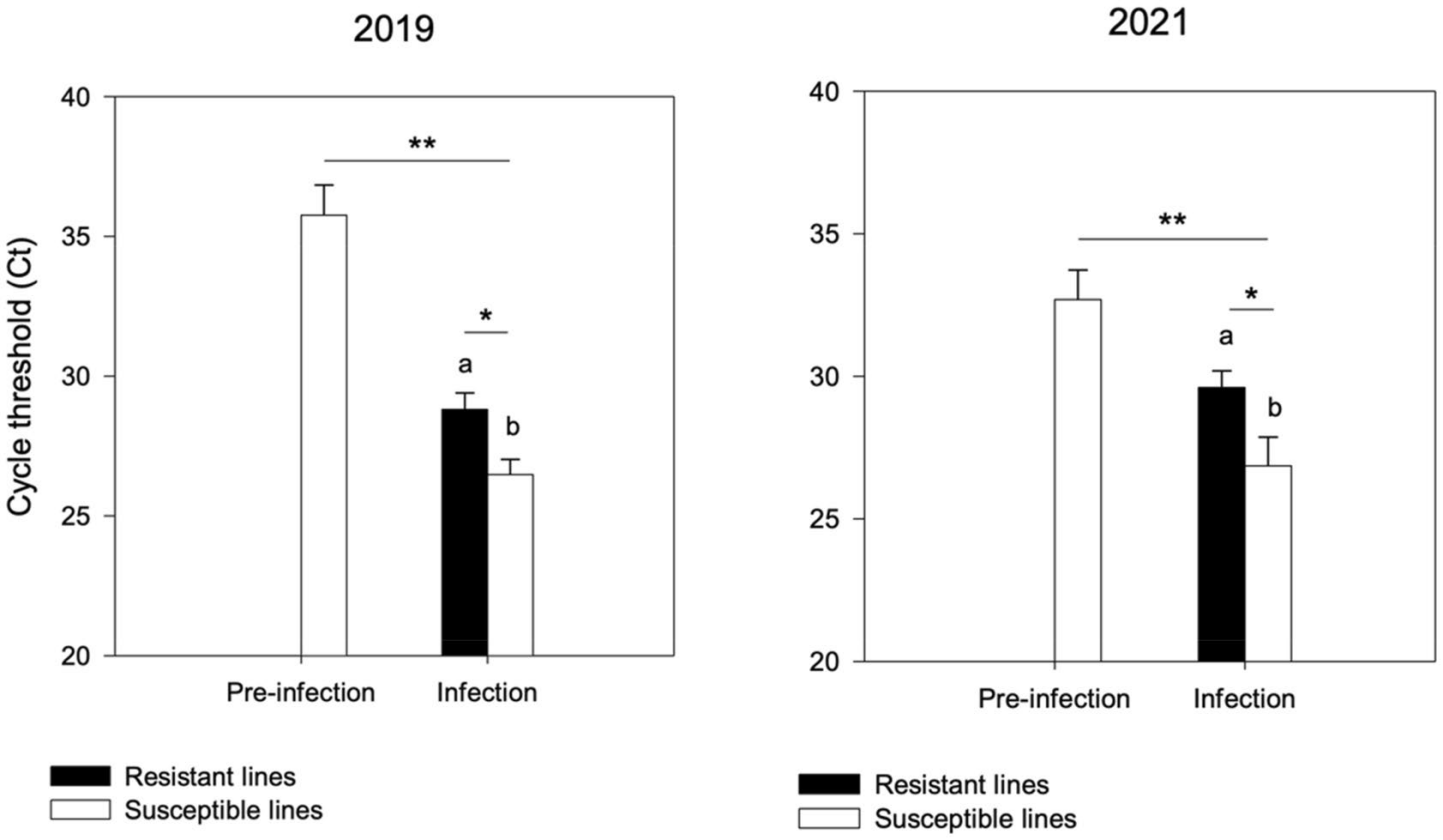
Relative abundance by qPCR of Cercospora beticola in susceptible sugar beet genotypes under pre-infection and infection resistant and susceptible sugar beet genotypes under infection stage. One asterisk and two asterisks indicate significant differences at p < 0.05 and p < 0.01, respectively (generated using SigmaPlot version 11.0, www.systatsoftware.com).

To ascertain and quantify the presence of CLS within 209 sugar beet individuals grown in field conditions, we also targeted the *Cercospora beticola* rDNA by qPCR (Fig. 4) on the same set of samples under pre-infection and infection stages. Under the pre-infection condition, we detected PCR-positive individuals only on samples from the susceptible line (Fig. 4). A significant difference in the abundance of the fungus was detected in the susceptible genotypes under infection compared to pre-infection (p < 0.01). As expected, under infected conditions, the fungus was detected in all samples of both resistant and susceptible lines. Particularly, a higher abundance (p < 0.05) was found in the susceptible genotypes with respect to the resistant ones for the years 2019 and 2021. Digital PCR-based quantification is shown in Supplementary Fig. S5.

**Figure 5:**
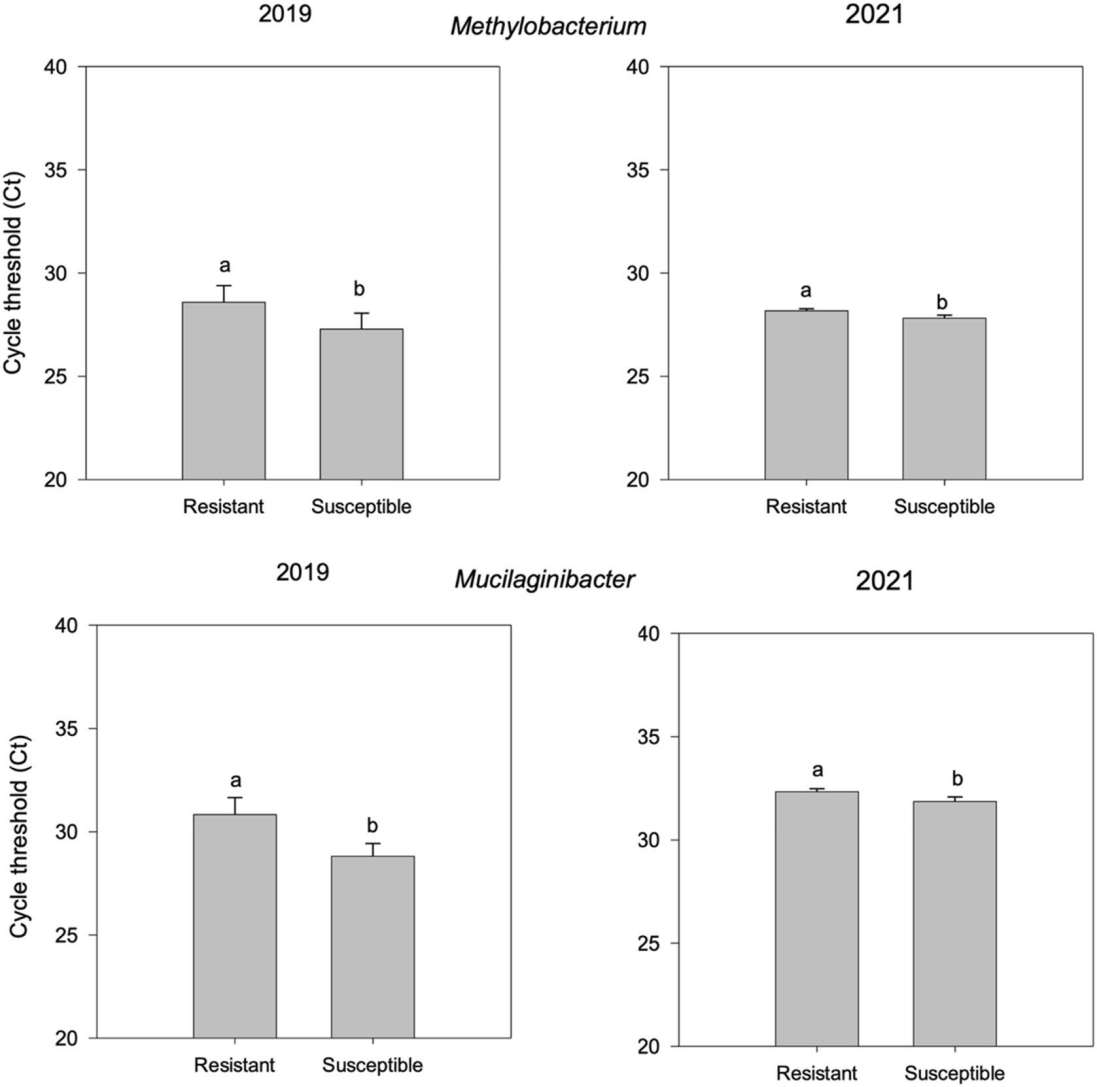
Relative abundance by qPCR of *Methylobacterium* (top) and *Mucilaginibacter* (bottom) in resistant and susceptible cultivated sugar beet genotypes under *Cercospora* infection stage. Plots were generated using SigmaPlot version 11.0, www.systatsoftware.com.

To verify these data are relevant for field-grown sugar beet, we targeted *Methylobacterium* and *Mucilaginibacter* within the same 209 sugar beet individuals collected under the infection stage. The results reported in Fig. 5 confirm the pattern of abundance of endophytic species assessed by qPCR in sugar beet individuals under CLS infection conditions. *Methylobacterium and Mucilaginibacter* showed significantly higher abundance (p < 0.05) in susceptible samples compared to resistant ones. *Methylobacterium* genus showed an average Ct value of 28.58 ± 0.81 and 28.17 ± 0.1 in resistant samples and 27.2 ± 0.77 and 27.8 ± 0.15 in susceptible samples for the years 2019 and 2021, respectively. *Mucilaginibacter* showed an average Ct value of 30.83 ± 0.82 and 32.33 ± 0.15 in resistant samples and 28.81 ± 0.61 and 31 ± 0.22 in susceptible ones for the years 2019 and 2021 respectively.

## Discussion

The occurrence of a given leaf microbiome is related to both the host genetics and environmental conditions. In some species, denser populations of microorganisms have been found in leaf areas more prone to the entry of microbes, such as stomatal opening and nearby leaf veins^26^. Damaging ultraviolet radiation, low levels of water, and nutrients can be stressors for microorganisms causing them to seek refuge inside plants^27^. In the same way, a plant, when undergoing stress, such as drought, nutritional limitations, herbivore damage, or the invasion of pathogens can seek help from microbes by fostering their entrance as endophytes recruiting them at higher levels. However, the success of endophytes in protecting the plant depends on the severity of the stress and the genotypic resources of the plant itself^28, 29^. It is therefore expected that weaker plants including those that, due to more susceptible genotypic configurations and suffering pathogenic injury would more actively attempt to cope with the stress by invoking microbial endophytes^30^.

In this work, we found substantial differences in the leaf microbiome composition when comparing symptomless and symptomatic sea beets. Particularly, *Alphaproteobacteria* and *Gammaproteobacteria*, two large classes of the phylum *Proteobacteria* were found to be significantly differentially enriched. Their abundance has also been reported in the literature as pre-dominant groups normally present in the leaf microbiome of plants^31, 32^. Notably, among *Alphaproteobacteria,* we found the genera *Sphingomonas, Methylobacterium*, and *Pseudomonas*. These three genera accounted for more than 70% of the sequences found through sequencing. Importantly, all three species were significantly more abundant in plants infected by CLS. Other bacteria found less frequently but still significantly overrepresented in the symptomatic plants were from the phyla of the *Actinobacteria* (*Propionibacterium*) and *Bacteroidetes* (*Mucilaginibacter*). Conversely, the members of the classes *Betaproteobacteria* (*Massilia*) and *Gammaproteobacteria* (*Pseudomonas)* were low in overall counts and not significantly different in relation to CLS disease.

The presence of *Sphingomonas* was observed in more than 63% of the samples. This gram-negative bacterium has been studied for its role in environmental remediation owing to its ability to bind heavy metals and enhance the expression of cysteine-rich metallothionein proteins^33^. The *Sphingomonas* genus, as an endophyte also has an important role in counteracting biotic and abiotic stresses, such as mitigation of salinity stress^34^ and protection against leaf-pathogenic *Pseudomonas syringae* and *Xanthomonas campestris*^35^*.* Particularly, *Sphingomonas* and *P. syringae* are direct competitors for glucose, fructose, and sucrose. The possible plant-protecting effect of *Sphingomonas* may be due to their high abundance on the leaf surface since early colonization is an important determinant for an effective biocontrol agent^36^. Its occurrence in plants calling for increased defense is congruent with the plant protective effect of *Sphingomonas* reported by Innerebner et al.^35^.

The above differences suggest also that some of the inner bacterial dwellers of *Beta* plants could be better suited to offer defense mechanisms sought for by the plant. In this sense, *Methylobacterium* appears as the genus that is most significantly differentially abundant between symptomless vs symptomatic and resistant vs susceptible individuals. Besides, it was found to be one of the predominant endophytes from sequencing (22% of the overall sequences). While many endophytes are known to enter from the root apparatus, some can access plants via stomatal openings. In this respect, it is worth remarking that the main ecological niche of the genus *Methylobacterium* is the phyllosphere. They are typically considered the most abundant bacterial genera ranging between 10^4^ and 10^7^ colony-forming units per gram fresh weight^37, 38^. Strains of *Methylobacterium* have also been found to improve potato yield under adverse conditions^39^. Another study showed that the *Methylobacterium* genus fostered plant growth through auxin and cytokine biosynthesis^40^.

While plants seeking protection via endophytic admittance are aimed at higher defense responses, this strategy does not guarantee success in disease avoidance as it is largely influenced by the plant’s own genotypic background. This is shown in tomato where *Methylobacterium* has been found to affect the physiological condition of the plants either positively or negatively^32^. Therefore, as mentioned, the strategy of endophytic recruitment and intensification is not in itself a measure that ensures a guaranteed biocontrol strategy. At this stage, we do not know which other possible covariates could be driving this phenotypic response. Further studies are envisaged to add details on this matter.

Another genus with enhanced representation in CLS infected leaves was *Mucilaginibacter* which is known to be rhizosphere associated endophyte in many plants like *Arabidopsis thaliana*, *Lotus parviflorus*, *Trifolium pratense* and *Fragaria x ananassa*^41–44^. It has been described to have roles in plant growth promotion^45, 46^. In one of the studies, it was observed that *Mucilaginibacter* as an endophyte was shown to alleviate salt stress in *Arabidopsis*^41^. In a very recent and interesting report, a greater relative abundance of *Mucilaginibacter* in *Verticillium dahliae*- and *Macrophomina phaseolina*-infested strawberry cultivars was shown, coherent with our observation^44^.

In this report, the correlation between different species of endophytes concentration and CLS occurrence has been shown both by examining spontaneously growing populations of *B. maritima* scattered across 15 locations in different countries, and subsequently confirmed in cropped plants using both resistant and susceptible genotype varieties during the CLS infection. The gradient of bacterial target detectability, unfolding in precise agreement with both the disease progression and host susceptibility (Figs. 2, 3, 4, and 5) is supportive of the phenomenon. In interpreting these data, one hypothesis could be that, since CLS development coincides with the progression of summer, the higher content of endophytes could just be part of a general increase in overall plants as a mere seasonal trend. However, this might not be the sole possibility explaining the observed data since endophytes increase in sugar beet breeding lines grown under field conditions is also differentially and statistically higher in the susceptible line when compared at a comparable stage with the resistant variety and thus the latter was indeed effectively less impacted by CLS infection.

Regarding technical considerations on the approach that was followed in this study, we first defined taxa that were consistently featured within the endophytic microbiome of the plants under investigation using by NGS 16 s amplicon sequencing. Having acquired that information, we targeted the relevant candidates using quantitative PCR and the same DNA extraction method exploiting the possibility to work simultaneously on large numbers of samples, as in the case of the QuantStudio 12 K Flex, which can process up to 12,000 qPCR samples in the same run. These automated DNA extraction and purification technologies enabled high processivity to the screening and a robust statistical design. As an additional check, we finally explored the performance of digital PCR, which could be recommended in cases where no signal arises from regular qPCR. The sensitivity of digital PCR is 100 -fold higher due to individual amplifications in distributed segments of the chip and specific target-annealing oligonucleotide probes based on TaqMan technology. Additionally, digital PCR is recommended for the absolute quantification of a specific low abundant target^47^ and to establish the concentration of a reference target for subsequent use in other platforms like qPCR^48^. This relatively novel technology has been used to quantify *Aspergillus* species in soils collected from raisin vineyards^49^ and to enumerate probiotic strains of *Lactobacillus acidophilus* and *Bifidobacterium animalis* replacing the traditional plate counts because of its extreme precision^50^. In this study, the use of digital PCR has therefore served as a double-check of already obtained data by qPCR based on flanking primers-directed amplification. This technique could be recommended as an alternative to standard qPCR in cases where the abundance of target organisms would be too low to be detected by Real-Time PCR (e.g. those yielding undetermined Ct outputs).

In conclusion, the abundance of a defined species within the plant endophytic microbiome is strongly correlated with a major physio-pathological condition of the plant, in this case specifically with plants infected by *Cercospora*. This evidence is interpreted as consistent with the notion that stressed plants more crucially seek the help of potentially beneficial microorganisms to increase their chances to cope with the disease. Plants facing environmental abiotic or biotic stresses are known from literature reports to be richer in endophytic taxa compared to unstressed controls. The admittance of endophytes however is not in itself a guarantee that the host would remain disease/stress-free. On the contrary, just like in our healthcare situations, an activated immune reaction, or the fact of undergoing therapy is consistent with the presence of the disease and not with its avoidance. Therefore, an increased load of endophytes is not expected to be directly associated with resistant plant genotypes since such plants need not rely on the trade-off deals with external helpers. What we are observing here suggests that plants that are more prone to CLS disease and loosening barriers for endophyte recruitment, are those which have the lowest level of effectiveness in their genotypic potential of resistance to the pathogen. Therefore, we propose that an assay of endophytic abundance, especially for the genera which resulted differentially featured in symptomless vs symptomatic beet plants (e.g. *Methylobacterium* and *Mucilaginibacter*), can be routine screened to serve the needs of breeders seeking markers associated with disease resistance for assets of the commercial seed production in worldwide sugar beet cropping.

## Material and methods

### Sea beet sampling for sequencing and microbiome analysis

**Figure 6:**
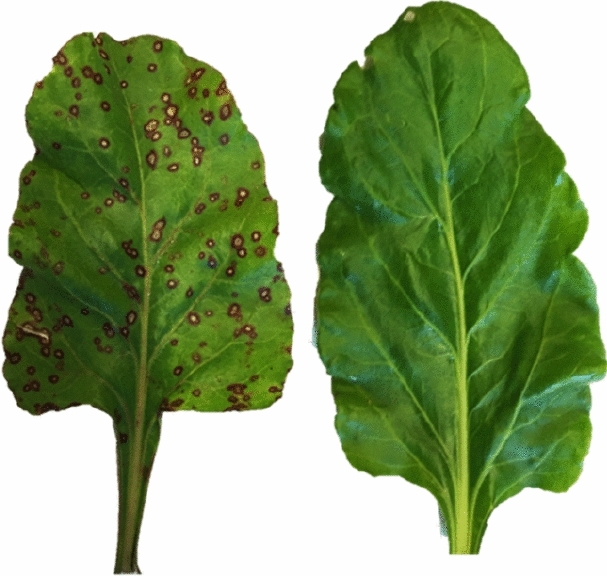
An example of CLS symptomatic leaf to the left and symptomless leaf to the right.

Samples from sea beet used for sequencing analysis were collected in Palmižana (Croatia) and Torcello (Venice, Italy) in August 2018. For each of the two locations, seeds were collected from clearly symptomatic (Palmižana) and symptomless (Torcello) plants (Fig. 6).

Seeds were then sown at the experimental farm at the University of Padova (Legnaro, Italy), in March 2019, to confirm their phenotypic status under natural field conditions. We selected a field with two crop-rotation (sugar beet-wheat) and endemic *Cercospora beticola* infection which was validated with a soil-based PCR test to ascertain the presence of the fungus. True phenotypes of 60 plants were evaluated between March and August 2019 in Padova, Italy. The experimental design was divided into four randomized blocks, each one divided into four subplots of 2.7 × 10 m dimension. Outside the randomized block, a control plot was placed, and plants were maintained without any treatments. Four leaves from symptomless and four leaves from symptomatic plants were then collected in August 2019. Samples were placed in sterilized 2 ml Eppendorf tubes and carried immediately to the laboratory for DNA extraction. Samples were not frozen to avoid artifacts in the resulting microbiome composition upon metabarcoding^51^.

### DNA extraction

For metabarcoding and sequencing, DNA was extracted from four CLS symptomatic (Palmižana) and four CLS symptomless (Torcello) sea beet leaves. The same method described below was used to extract DNA for the quantitative PCR analysis on the 504 specimens subsequently collected for validation in the years 2019 and 2021 and for the digital PCR validations (described later). The leaf surface of sea beet leaves was sterilized by immersion for 3 min in 0.5% sodium hypochlorite, 3 min in 70% ethanol, and rinsed in sterile deionized water. 50 mg of fresh leaf material were homogenized using a Tissue Lyser (Qiagen, Hilden, Germany) for 5 min at 30 Hertz in a 2 ml Eppendorf tube with 300 μl of RTL buffer (Qiagen). Samples were centrifuged for 5 min at 6000×*g*, after which the supernatant was collected. DNA purification with Biosprint 96 involved the use of five S-block plates and one flat 96-well plate. The first S-block plate contained 300 μl of the sample (supernatant) together with 200 μl of isopropanol and 20 μl of MagAttract magnetic beads suspension (Qiagen). The second plate was filled with 500 μl of RPW buffer and the third and fourth with 500 μl of ethanol (96%). The fifth S-block plate contained 500 μl of tween solution at 0.02%. DNA was diluted in a flat 96-well plate with 100 μl of nuclease-free water. DNA was quantified with a Qubit 3.0 Fluorometer (Thermo Fisher Scientific, USA) using the Qubit DNA HS Assay Kit Fluorometer (Thermo Fisher Scientific, USA).

For the DNA extraction from soil samples to ascertain the presence of the fungus Cercospora, the method published by Chiodi et al. 2020^52^ was used.

### Sequencing and bioinformatics data analysis

A 16S Ion Metagenomics Kit (Thermo Fisher Scientific, USA) was used to amplify seven hypervariable regions of the 16S rRNA genes. Regions V2, V4, V8 and V3, V6-7, V9 were amplified in two separate PCR reactions. The amplification program was set as follows: 95 °C for 10 min, 25 cycles of 95 °C for 30 s, 58 °C for 30 s, 72 °C for 20 s, and a hold stage at 72 °C for 7 min. Amplicons were pooled together to have a final concentration of 30 ng μl^−1^ for the library preparation with Ion Xpress Plus Fragment Library Kit (Thermo Fisher Scientific, USA). Each sample was ligated with a unique barcode using the Ion Xpress Barcode Adapter (Thermo Fisher Scientific, USA). Libraries were amplified with the following program: 95 °C for 5 min, 7 cycles of 95 °C for 15 s, 58 °C for 15 s, and 70 °C for 1 min, then stored at 4 °C. A Qubit 3.0 Fluorometer with Qubit DNA HS Assay Kit was used to quantify the libraries. 25 pM of pooled libraries were processed with One-Touch 2 and One-Touch ES system reagents (Thermo Fisher Scientific, USA) following the manufacturer’s instructions. Sequencing was performed with Ion GeneStudio S5 System on Ion 520 chip using 850 flows for 400-bases-read sequencing.

Base-calling and run demultiplexing were performed by Torrent Suite Software version 5.10.0 (Thermo Fisher Scientific, USA) with default parameters. Ion Reporter cloud software version 5.12 (Thermo Fisher Scientific, USA), was adopted to process 16S metagenomic data using default parameters. Taxonomic assignment of unique reads was done using a multi-stage BLAST with the Greengenes v13.5^24^ and MicroSEQ 16S reference libraries v2013.1 which in-built databases on Ion Reporter (Thermo Fisher Scientific, USA). At this stage, care was taken to identify and remove reads assigned to chloroplast and mitochondria. To strengthen and support the preliminary analysis using Ion Reporter (Thermo Fisher Scientific, USA), an alternative pipeline was also used to process the data. First, the raw reads were trimmed for 20 base pairs on both ends to remove primers using cutadapt^53^ and analysed using QIIME2 v2020.08^25^ pipeline. Imported reads were then denoised and dereplicated using qiime dada2 plugin. This was followed by taxonomic classification of ASVs by classify-consensus-blast plugin using Silva SSU v138.1^54^ as the reference database. The taxonomy abundance table at genus level was further processed using DESeq2^55^ to normalize for the library size. The resultant normalized taxonomy table was filtered for taxas for average reads resulting greater than 10 when combining all samples and used for comparison between the symptomatic and symptomless phenotypes. Taxonomic abundance plots were made using ggplot2 package in R^56^. PCA-biplots were generated using the FactoMineR package in R^57^ to visualise the contribution of the most abundant genera to the sample phenotypes. The abundance matrix was also used to develop diversity plots using Calypso web tool^58^. LDA Effect Size (LEfSe) analysis^59^ was performed on the abundance matrix obtained from QIIME2. The genera resulting differentially abundant were considered as targets for downstream validation using qPCR to correctly ascertain the association of the bacteria to the phenotype.

### Primer design for PCR-based validation

The 16S rRNA sequences of the seven most recurring and differentially abundant bacterial taxa were used to design primers to be used in quantitative PCR. The ribosomal DNA sequence of *Cercospora beticola* (NCBI accessions MF681167.1, MF681115.1, and AY840527.2) was also used to design qPCR primers. The software Primer Express v3.0 (Thermo Fisher Scientific, USA) was used to pick the suitable forward and reverse oligomers. The primer pair sequences and the corresponding targeted bacteria are shown in Table 2**.**

**Table 2:** Primer pairs used in this study for quantitative PCR analyses.

Endophytic bacteria	Primer forward 5ʹ, 3ʹ	Primer reverse 5ʹ, 3ʹ
*Methylobacterium*	CTTCCGGTACCGTCATTATCG	GTGATGAAGGCCTTAGGGTTGT
*Mucilaginibacter*	TCCGGATTTATTGGGTTTAAAGG	ACCGTCTTTCACCCCTGACTT
*Cercospora beticola*	TGAGGGCCTTCGGGCT	ACTCCGACGCAAAGATGCAGT

### Sea beet sampling for qPCR validation from spontaneous plant populations

**Figure 7:**
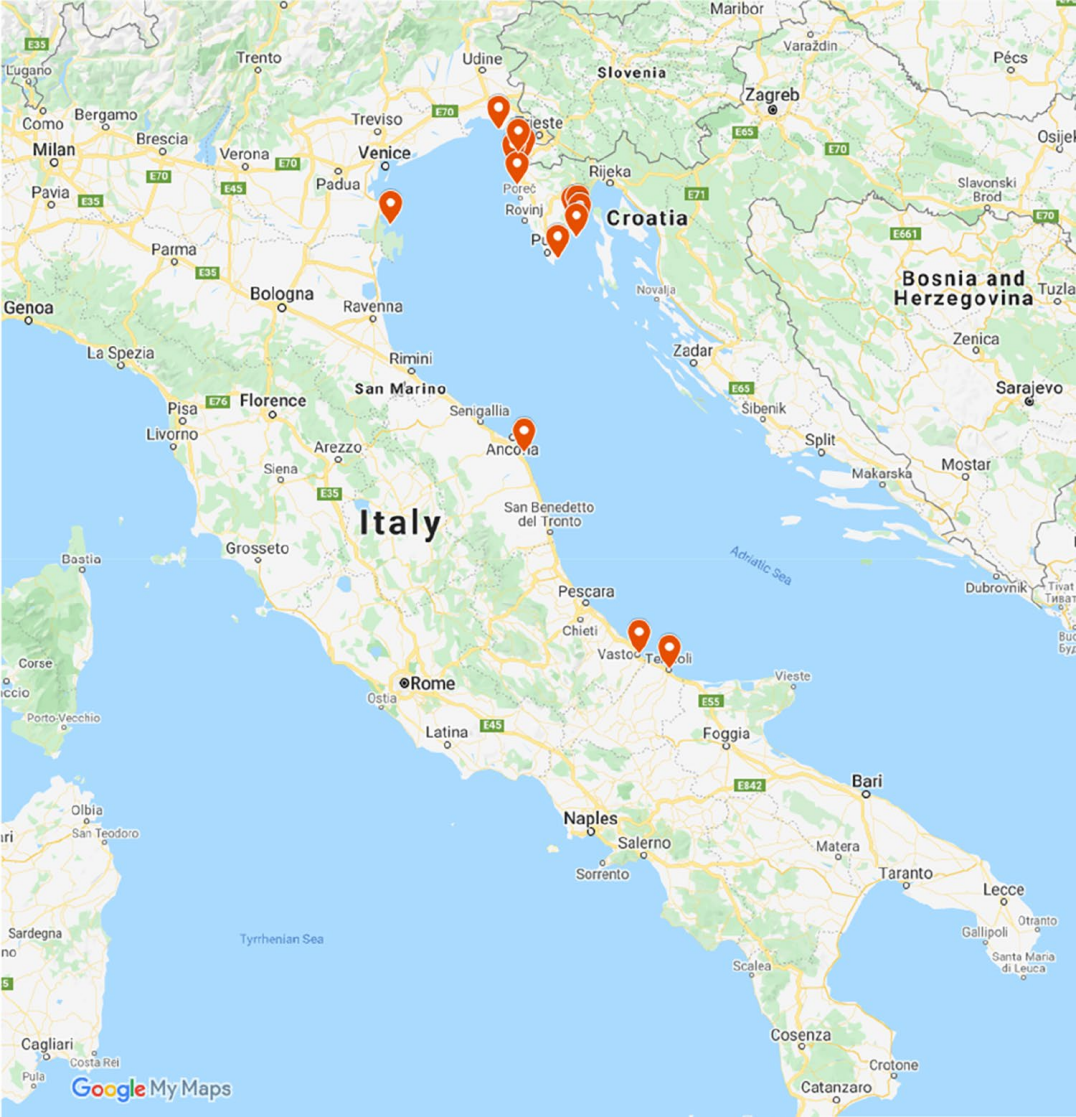
Locations spanning the Mediterranean coast from which the 504 naturally occurring plant specimens of sea beet were collected are pinned in red flags (Map data 2022 Google, https://www.google.com/maps).

The subsequent sampling campaigns to gather material to be analyzed by quantitative PCR targeting specific bacteria were carried out in 2019 and 2021. 302 sea beets (145 with symptomatic leaves and 157 symptomless leaves) were collected in 2019 (Fig. 7) in one-month time frame (July 2019). 166 sea beets (71 with symptomatic leaves and 95 symptomless leaves) were collected in 2021 (Fig. 7) in one month time frame (July 2021). Table 1 reports the locations and number of samples collected from each site. Leaves were placed in a 50 ml Falcon tube, stored on ice, and transferred to the laboratory for DNA extraction. A map with all the sampling locations pinned is presented in Fig. 7 to visually appreciate the span of sampling.

### Leaf sampling for qPCR validation of sugar beet breeding lines grown under field conditions

A total of 120 sugar beet leaf samples derived from two susceptible and two resistant parental lines of Strube Research GmbH & Co. KG were collected in 2019. A total of 89 sugar beet leaf samples derived from one susceptible and two resistant parental lines and five susceptible and seven resistant hybrids of Strube Research were collected in 2021. All samples were collected in a field trial conducted by Carla Import Sementi SRL at Rovigo (Italy). The field trials were laid out in a randomized incomplete block design with four replicates. Seeds were sown in two rows plots on March 6, 2019, and March 3, 2020. Each plot consisted of an additional third row sown with a hybrid susceptible to CLS to facilitate the natural fungal infection in the field. The scoring of CLS was performed per plot during the growing season of the plants using a scale from 1 (no infection) to 9 (entire defoliation)^60, 61^. The same described genotypes and plant material were collected before the onset of CLS infection on June 12, 2019, and June 14, 2021, and under CLS infection on August 8, 2019, and August 9, 2021, for comparative analysis.

### Quantitative PCR

The presence of specific bacterial 16S rRNA sequences and that of *Cercospora beticola* targeting rDNA was tested by Real-Time qPCR with QuantStudio 12 K Flex (Thermo Fisher Scientific, USA) using the primers reported in Table 2 and Supplementary Table S1. The thermocycler program consisted of 10 min of pre-incubation at 95 °C, followed by 50 cycles of 15 s at 95 °C and 1 min at 60 °C. Reagent mixes were prepared by combining 5 μl of Sybr Green Real-Time PCR Master Mix, 0.1 μl (10 μM) of each primer, 1.4 μl of nuclease-free water, and 1 μl of the sample with a concentration of 2 ng/μl. All samples were analysed in triplicates.

### Digital PCR

For the highest levels of sensitivity, a quantitative PCR approach of digital PCR was performed with a QuantStudio 3D Digital PCR System (Thermo Fisher Scientific, USA) to validate the Real-Time PCR data on selected samples and obtain further quantitative information. Quantitative TaqMan assays were developed based on 16S rRNA sequencing results in the V2 region of the bacterial sequences obtained by Ion Reporter cloud software. *Cercospora* quantification was also done using dPCR (Supplementary Figs. S4, S5). The PCR mix contained 8 μl of QuantStudio 3D Digital PCR Master Mix v2, 1.44 μl (10 μM) of forward and reverse primers, 0.40 μl of FAM probe, and 3.22 μl of nuclease-free water. Chips were loaded with 14.5 μl of mix and 1.5 μl of DNA at a concentration of 10 ng μl^-1^. Amplification was performed on a ProFlex Flat PCR system (Thermo Fisher Scientific, USA) with the following thermal profile: 96 °C for 10 min, 40 cycles at 60 °C for 2 min, 98 °C for 30 s, and a final step at 60 °C for 2 min. Data were analyzed with a QuantStudio 3D Digital PCR Analysis Suite Software v3.0 (Thermo Fisher Scientific, USA).

### Statistical analyses of qPCR results

The Ct values of all sample groups (symptomless and symptomatic for sea beets; resistant and susceptible for cultivated beets) were subject to Maximum Likelihood (REML) analysis for estimating the variance components in TIBCO Statistica, 2020 (https://www.tibco.com/). Wald-F values were computed for fixed effects to determine statistical differences between the categorical variables for *Cercospora* data and the two bacteria *Methylobacterium* and *Mucilaginibacter*. The normality distribution of the residuals was verified with both Shapiro–Wilk test and residuals plots. Homogeneity of variances was checked with Levene’s test. Statistical significance of differences was assessed by hierarchical Restricted Maximum Likelihood (REML) variance component analysis. Post-hoc analysis for pairwise comparisons was carried out by the Bonferroni test. The plots were developed using SigmaPlot version 11.0 (SYSTAT, San Jose, CA, USA).

### Declaration for plant sampling

The sampling of plants was carried out following the Regulation (EU) 2016/2031 on protective measures against plant pests (“Plant Health Law”), and all permissions needed to collect "leaf samples" of *Beta vulgaris* L. were obtained.

## Supplementary Information

Supplementary Information.

## Data Availability

The datasets generated during the current study are available from the corresponding author on reasonable request.
