# Supplementary material for: Bacterial endophytes as indicators of susceptibility to Cercospora Leaf Spot (CLS) disease in Beta vulgaris L
Source: Sci Rep. 2022 Jun 23;12:10719. doi: 10.1038/s41598-022-14769-8 (PMC9226160; doi:10.1038/s41598-022-14769-8)
Supplement: Supplementary file 1 — Supplementary Information. [file 41598_2022_14769_MOESM1_ESM.docx]

**
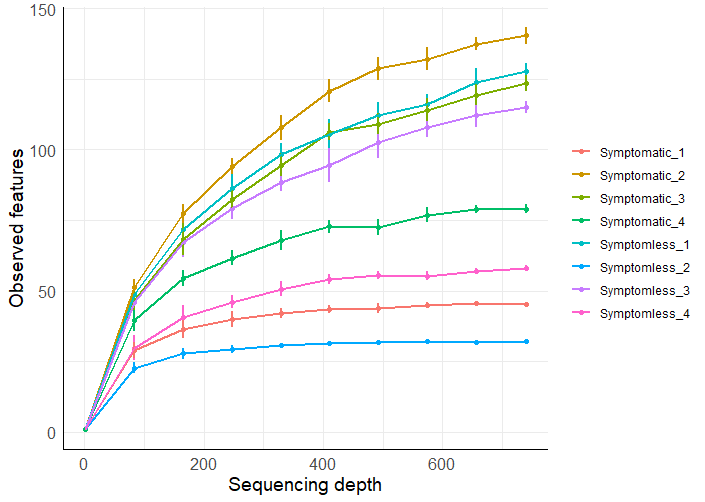
**

**A**


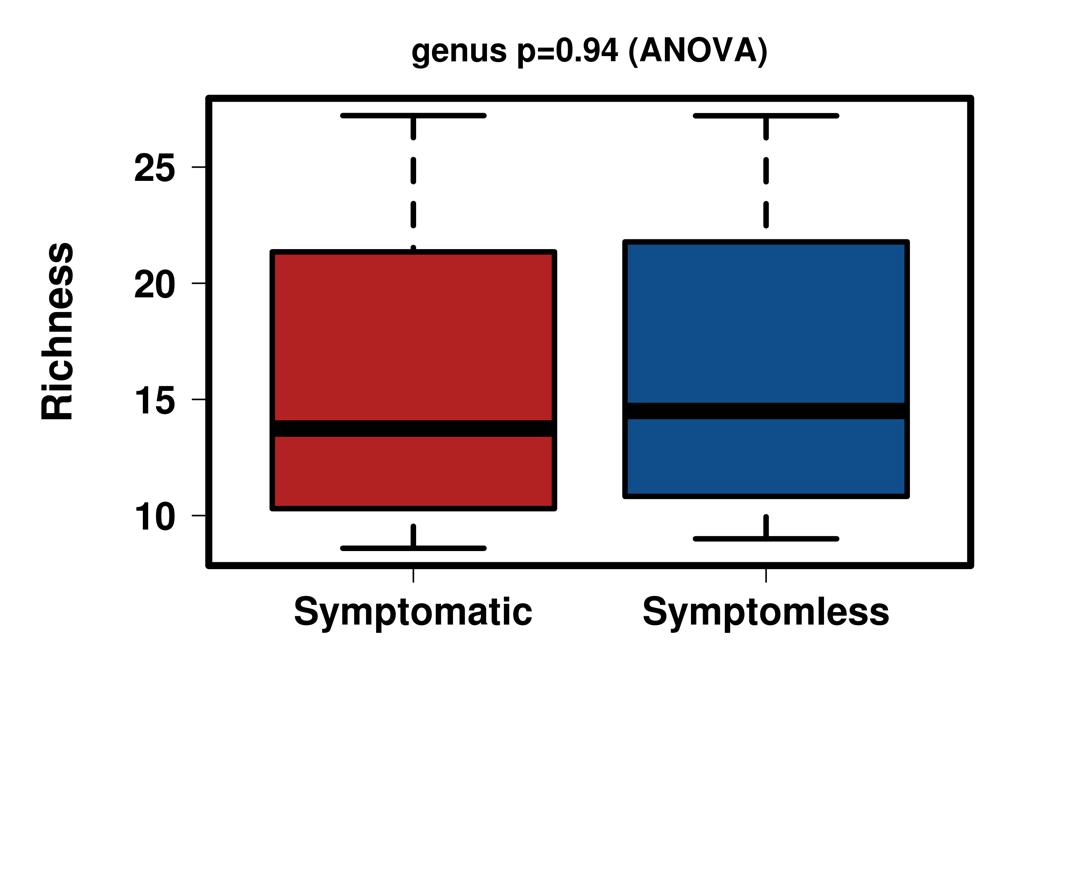


**B**

**Supplementary Figure S1: A) Rarefaction curves reporting the number of reads sampled on the X-axis for each analyzed sample and the number of observed species on the Y-axis. Each curve represents a single sample. B) Alpha diversity assessed using Richeness as index showed no significant difference in the count of the number of different species present among and between the two sample groups (p value=0.97)**


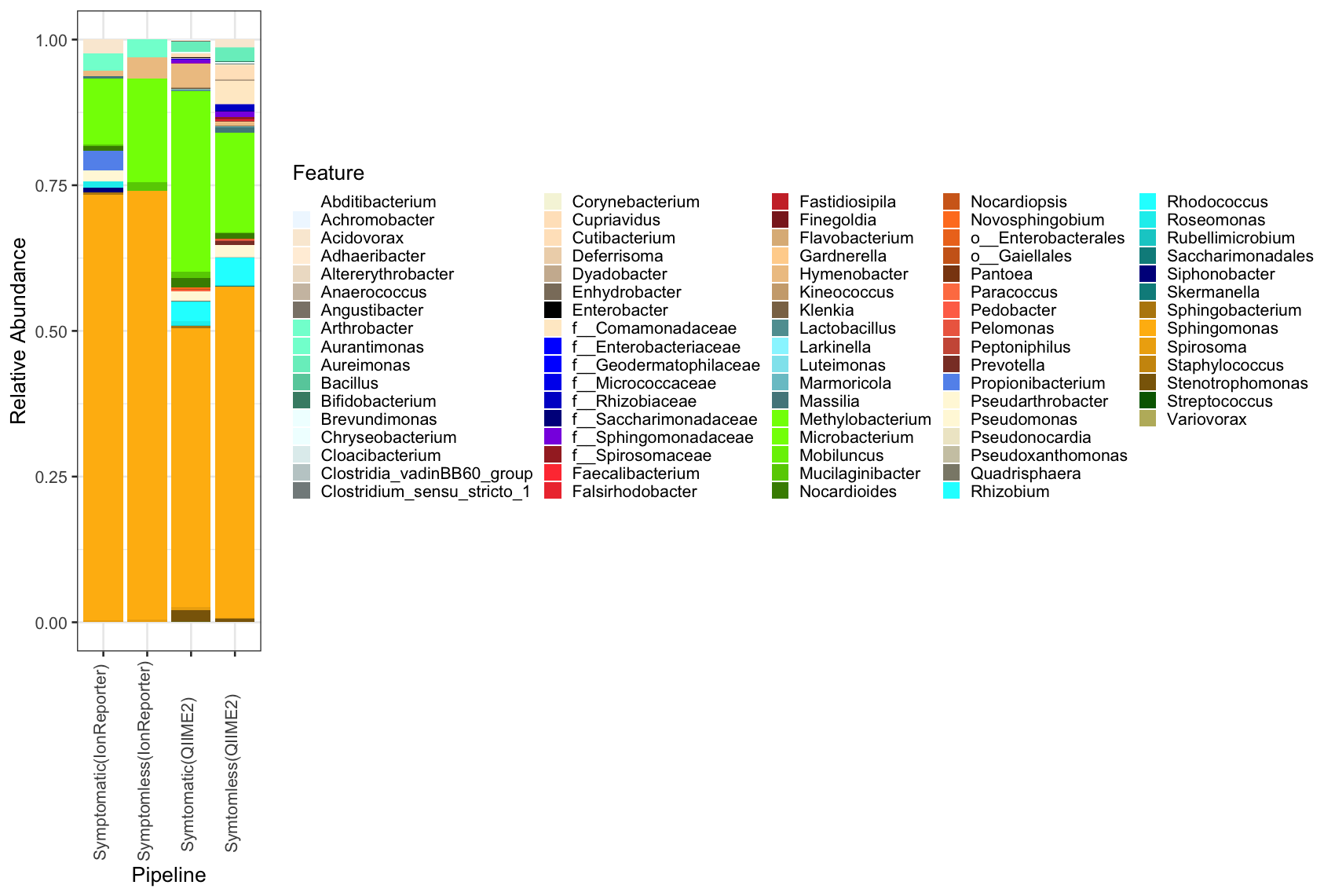


**Supplementary Figure S2: Comparative analysis from IonReporter and QIIME2 pipeline showing conservation of major taxa between symptomless and symptomatic plants. While the IonReporter method does OTU based clustering, QIIME2 utilises the ASV approach for clustering reads.**

**
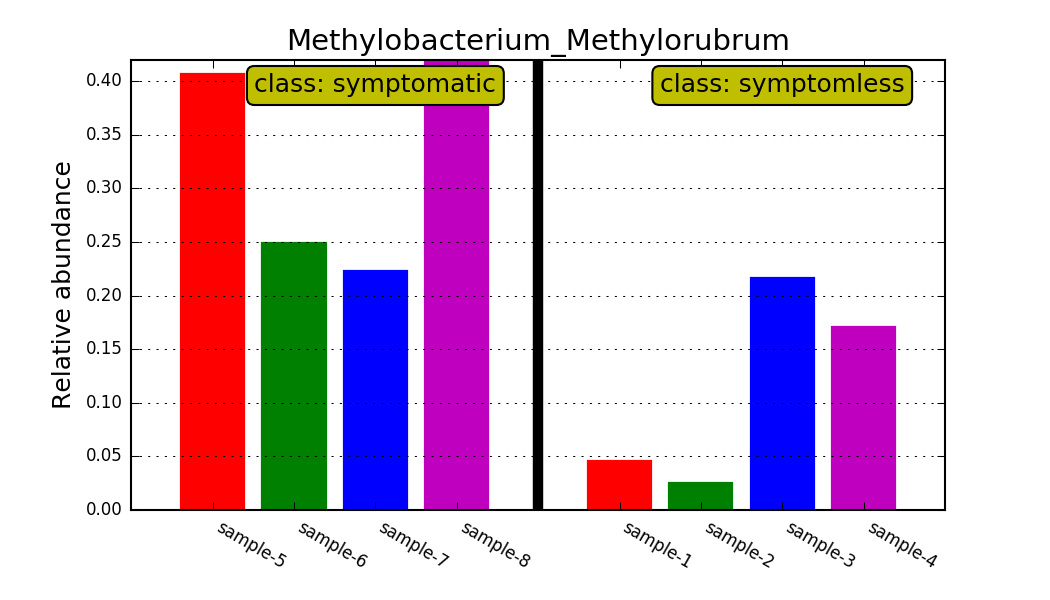
**

**A**

**
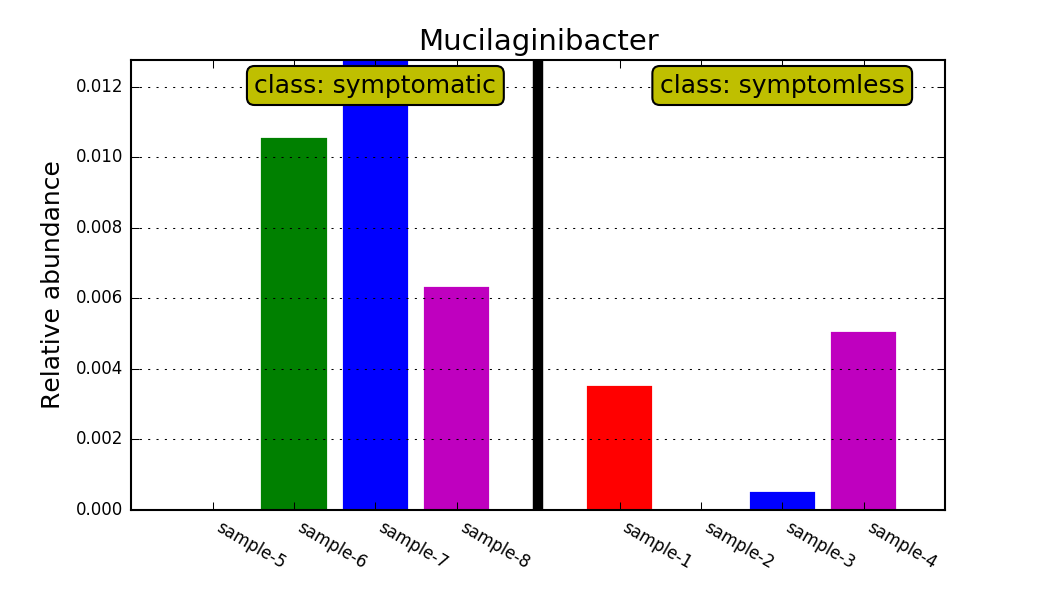
**

**B**

**Supplementary Figure S3: Differential relative abundance of A) *Methylobacterium* and B) *Mucilaginibacter* of features detected by LEfSe as biomarkers.**

**
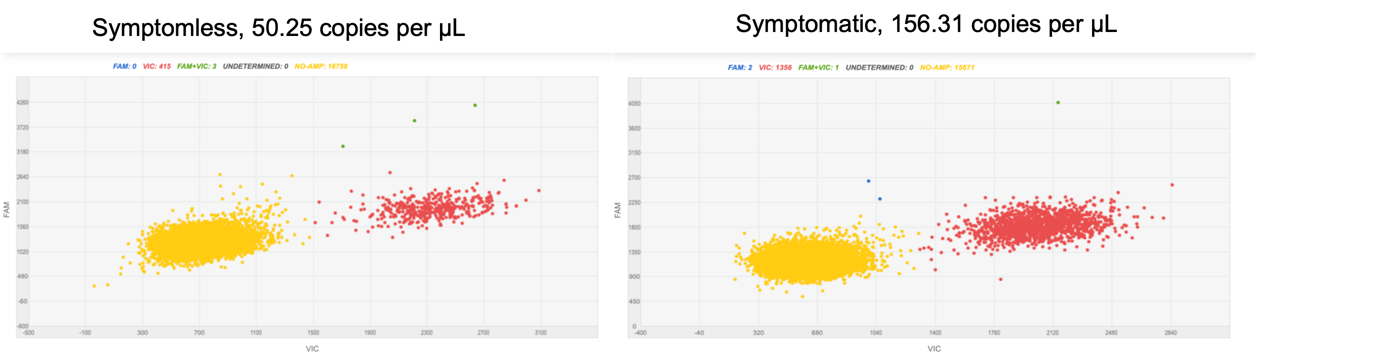
**

**Supplementary Figure S4: Digital PCR based absolute quantification of *Cercospora beticola* on symptomless and symptomatic sea beets (QuantStudio™ 3D Digital PCR Analysis Suite™ Software v3.0, Thermo Fisher Scientfic, USA)**

**
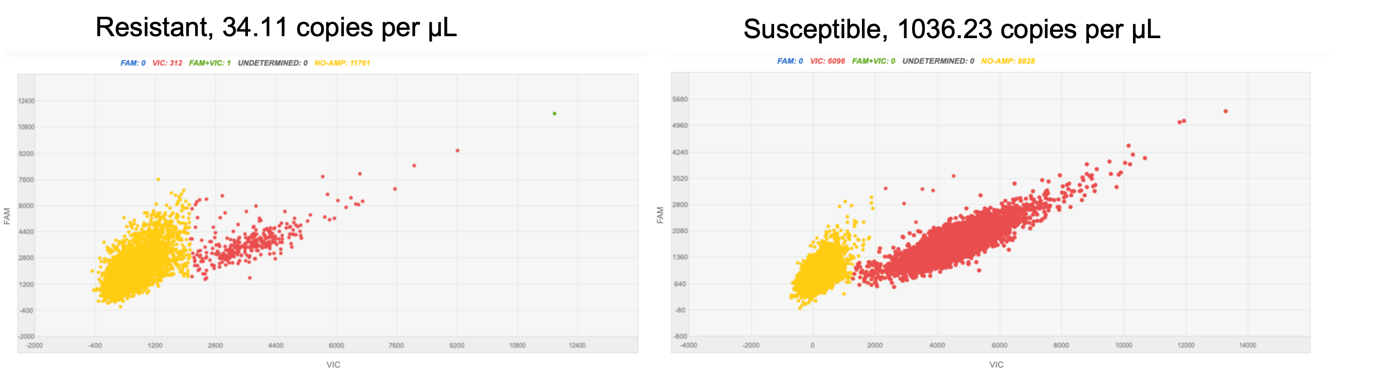
**

**Supplementary Figure S5: Digital PCR based absolute quantification of *Cercospora beticola* on resistant and susceptible beets under infection stage (QuantStudio™ 3D Digital PCR Analysis Suite™ Software v3.0, Thermo Fisher Scientfic, USA)**

| **Endophytic bacteria** | **Primer Forward 5’,3’** | **Primer Reverse 5’,3’** |
| --- | --- | --- |
| *Sphingomonas* | TGGGCGCAAGCCTGAT | GCTTTACAACCCTAAGGCCTTCA |
| *Aureimonas* | CCGGAACGGCCTTTGAT | CGCAATTCCACTCACCTCTTC |
| *Pseudomonas* | GCGCGTAGGTGGCTTGATAA | GGATGCAGTTCCCAGGTTGA |
| *Massilia* | CAATGCCGCGTGAGTGAA | GAACCGTTTCTTCCCTGACAAA |

**Supplementary Table S1: Primers of other candidates from sequencing not resulting significant across larger sea beet and cultivated beet validations.**
